# A Rare Case of Septic Shock Secondary to Emphysematous Hepatitis

**DOI:** 10.1155/2017/3020845

**Published:** 2017-05-30

**Authors:** Khaled M. Nada, Ibrahim El Husseini, Mohammad E. Abu Hishmeh, Neerav S. Shah, Nadezhda Ibragimova, Riyad Basir

**Affiliations:** ^1^Lincoln Medical and Mental Health Center, 234 E. 149th St., Bronx, NY 10451, USA; ^2^Department of Pulmonary/Critical Care, Lincoln Medical and Mental Health Center, 234 E. 149th St., Bronx, NY 10451, USA

## Abstract

**Objective:**

To describe a case of emphysematous hepatitis which is a rare clinical entity, characterized by a fatal, rapidly progressive infection of the liver with a radiological appearance simulating emphysematous pyelonephritis and to help provide more data about the causative organisms and precipitating factors of this pathology.

**Data Sources and Synthesis:**

Relevant literature was reviewed and, to the best of our knowledge, there is limited data regarding the pathogenesis, causative organisms, and management of this condition.

**Conclusion:**

Emphysematous hepatitis is a rapidly progressive infection that can be fatal in the absence of appropriate therapeutic intervention. Initial clinical manifestations are usually subtle and thus high clinical suspicion is required for early diagnosis and management of this condition to help decrease the mortality rates.

## 1. Introduction

While intra-abdominal emphysematous infections involving a variety of abdominal organs have been widely recognized, few reported cases have described similar emphysematous changes occurring in the liver [1]. Emphysematous changes in the liver have typically been seen in clinical situations involving gas-forming bacteria (e.g., liver abscesses), following sphincterotomy, bowel infarction, and hepatic artery thrombosis after liver transplantation [2, 3]. Blachar et al. (2001) first reported a case of a 43-year-old diabetic patient who was found to have extensive replacement of the entire liver parenchyma by air with no evidence of abscess or liver lesion [4]. Since then, only a few case reports have discussed similar radiographic findings. Despite aggressive management, all reported cases with emphysematous hepatitis have led to fatal outcomes within 24 to 72 h of admission. We report a case of fulminant liver failure in the setting of emphysematous hepatitis and bacteremia, in a patient with a history of pancreatic cancer and liver metastasis.

## 2. Case Presentation

A 73-year-old woman was admitted to the Medical Intensive Care Unit (MICU) at Lincoln Medical Center, Bronx, New York, due to abdominal pain and altered mental status of two days' duration. Upon the onset of her symptoms, she presented to the Emergency Department at another hospital; at that time, she had normal lab results, her symptoms improved with supportive management, and she was subsequently discharged. However, her symptoms worsened on the next day and she presented to our hospital. The patient had a past medical history remarkable for pancreatic cancer and had Whipple operation done eight months prior to presentation with adjuvant chemotherapy. However, six weeks prior to presentation, hepatic and lung metastasis were detected and chemotherapy was stopped. The patient's medical history was also remarkable for chronic obstructive pulmonary disease (COPD), hypertension, chronic hepatitis C, and pulmonary embolism that was incidentally discovered seven months prior to presentation for which she was receiving therapeutic low molecular weight heparin.

On presentation, the patient was obtunded, oriented only to person, and hypoglycemic with undetectable glucose level by finger-stick measurement; she received a total of 150 g of glucose in 50% solution as intravenous bolus. However, there was no improvement in her mental status and hence she was intubated for airway protection and admitted to the MICU.

The patient was acutely ill in appearance, afebrile, and vitally stable. Examination was remarkable for scleral icterus, ascites, and right upper quadrant tenderness, and the liver and spleen were not palpable. Initial complete blood count showed leukocytosis with a white blood cell count of 22.3 × 10^9^/L and 96.7% neutrophils, anemia with hematocrit of 22.4% and hemoglobin of 6.4 g/dl, and thrombocytopenia with a platelet count of 77 × 10^9^/L. Schistocytes were not appreciated on the peripheral smear.

**Figure 1:**
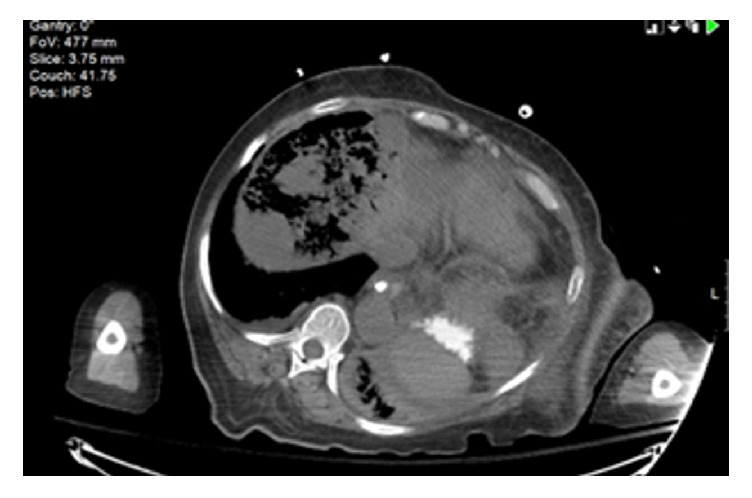
Parenchymal emphysema of the right hepatic lobe (transverse section).

**Figure 2:**
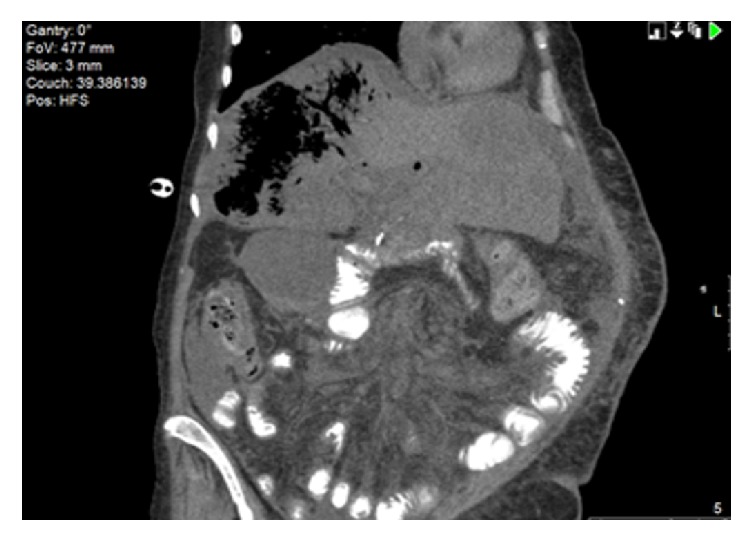
Parenchymal emphysema of the right hepatic lobe (longitudinal section).

**Figure 3:**
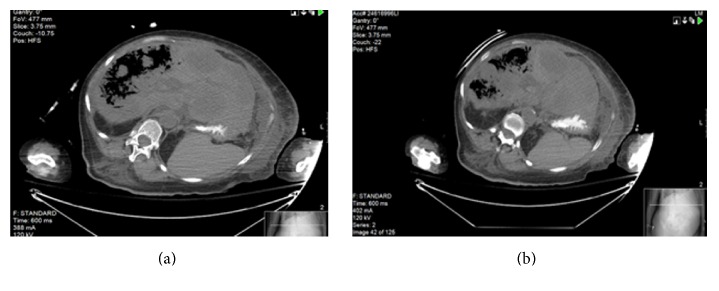
Showing hepatic metastasis spared by the emphysematous changes.

Blood urea nitrogen was 25 mg/dl and creatinine 1.92 mg/dl. Liver function tests showed aspartate aminotransferase (AST) of 7020 U/L, alanine aminotransferase (ALT) of 1435 U/L, alkaline phosphatase 305 U/L, total bilirubin of 9 mg/dl, and direct bilirubin of 4.4 mg/dl. Coagulation studies showed a prolonged prothrombin time (PT) of 37.8 seconds, with International Normalized Ratio (INR) of 3.28, and partial thromboplastin time (PTT) of 39.2 seconds. Ammonia level was 270 *µ*mol/L and arterial blood gas analysis after intubation on ventilator settings (AC/400 ml/20 bpm/100% FiO2/PEEP +5) was remarkable of high anion gap (AG) metabolic acidosis (AG 25) and metabolic alkalosis (pH 7.31; HCO_3_ of 17 mmol/L; PCO_2_ of 34.3 mmHg; PO_2_ of 440 mmHg; O_2_ sat. 100%; and lactic acid of 10.9 mmol/L). Brain CT scan was unremarkable. Abdominal CT scan demonstrated mottled gas opacity (Figures 1 and 2) throughout the parenchyma of the right hepatic lobe, sparing the hepatic masses (Figures 3(a) and 3(b)) with no focal air fluid collections.

Blood cultures were sent (grew pansensitive* Streptococcus mutans* and* Enterococcus faecalis*) and empiric broad-spectrum antibiotics were started (piperacillin + tazobactam). The patient also received one unit of packed red blood cells and three units of fresh frozen plasma, and surgical service was consulted for possible debridement, which was not attempted as her hemodynamic status rapidly deteriorated and few hours later she developed septic shock, requiring pressor support. Despite aggressive management, the patient expired within twenty-four hours after admission. The decision for autopsy was deterred due to lack of consent.

## 3. Discussion

Emphysematous hepatitis shares some radiological and clinical features with other similar entities such as emphysematous pyelonephritis which is an acute necrotizing, gas-forming infection of the kidneys that is fatal if not treated and usually associated with diabetes [5]. Usual bacterial pathogens associated with emphysematous infections (such as pyelonephritis) are* E. coli*,* Klebsiella*,* Enterobacter*,* Pseudomonas*,* Proteus*, and* Streptococcus* (the pathogen in this presented case) [6].

Emphysematous hepatitis is a very rare entity and only a few cases have been reported; initial clinical manifestations are usually subtle and progress rapidly in the absence of therapeutic interventions [1]. The pathophysiology of the emphysematous findings on imaging is due to bacterial infection causing mixed acid fermentation from tissue necrosis that ultimately forms nitrogen (60%), hydrogen (15%), carbon dioxide (5%), and oxygen (5%) and the impaired transport of these products of catabolism from the necrotic tissue [7, 8].

Other differential diagnoses for segmental collection of air in the liver parenchyma are pyogenic liver abscess (usually has fluid collection with possible gas formation) and hepatic infarction. Hepatic infarction is usually caused by damage of hepatic circulation (arterial/venous) from trauma or as a complication of liver transplant. The most feared cause of intrahepatic gas formation in a liver transplant is gangrene formation secondary to hepatic artery thrombosis [9].

Emphysematous hepatitis remains a rare entity with a significant poor outcome, since most patients with this severe infection do not improve with routine medical management (antibiotic therapy with or without drainage) and progress rapidly as evident in this case, when the patient presented to a different hospital one day prior to her ICU admission and at that time the patient had normal liver function tests and no evidence of sepsis. The diagnosis of this condition requires imaging preferably CT scan and the ability of clinicians/radiologists to suspect and identify the pathological process.

## References

[1] Grayson D. E., Abbott R. M., Levy A. D., Sherman P. M. (2002). Emphysematous infections of the abdomen and pelvis: a pictorial review. *Radiographics*.

[2] Doblecki-Lewis S., Palaios E., Bejarano P. A., Tzakis A. G., Selvaggi G., Morris M. I. (2008). Hepatic gas gangrene following orthotopic liver transplantation: Three cases treated with re-transplantation and a review of the literature. *Transplant Infectious Disease*.

[3] Johannes H., Westerkamp V., Trautwein C., Winkler M., Manns M. P., Hafer C. (2007). Hepatic gas gangrene following liver transplantation. *Liver Transplantation*.

[4] Blachar A., Federle M. P., Brancatelli G. (2002). Acute fulminant hepatic infection causing fatal "emphysematous hepatitis": case report. *Abdominal Imaging*.

[5] Lee H. M., Jeffrey R. B. (1995). Emphysematous pyelonephritis with resultant emphysematous cholecystitis secondary to hematogenous dissemination. *Abdominal Imaging*.

[6] Wan Y.-L., Lee T.-Y., Bullard M. J., Tsai C.-C. (1996). Acute gas-producing bacterial renal infection: Correlation between imaging findings and clinical outcome. *Radiology*.

[7] Schainuck L. I., Fouty R., Cutler R. E. (1968). Emphysematous pyelonephritis. A new case and review of previous observations. *The American Journal of Medicine*.

[8] Huang J.-J., Chen K.-W., Ruaan M.-K. (1991). Mixed acid fermentation of glucose as a mechanism of emphysematous urinary tract infection. *Journal of Urology*.

[9] Shaked A., McDiarmid S. V., Harrison R. E., Gelebert H. A., Colonna III J. O., Busuttil R. W. (1992). Hepatic artery thrombosis resulting in gas gangrene of the transplanted liver. *Surgery*.

